# Active surveillance of visual impairment due to adverse drug reactions: findings from a national study in the United Kingdom

**DOI:** 10.1002/prp2.107

**Published:** 2014-12-16

**Authors:** Phillippa M Cumberland, Isabelle Russell-Eggitt, Jugnoo S Rahi

**Affiliations:** 1 Life Course Epidemiology and Biostatistics Section, University College London (UCL) Institute of Child HealthLondon, United Kingdom; 2 Ulverscroft Vision Research GroupLondon, United Kingdom; 3 Great Ormond Street Hospital for Children NHS Foundation TrustLondon, United Kingdom; 4 National Institute for Health Research (NIHR) Biomedical Research Centre at Moorfields Eye Hospital NHS Foundation Trust and UCL Institute of OphthalmologyLondon, United Kingdom

**Keywords:** Active surveillance, adverse drug reactions, ocular, pharmacoepidemiology, visual impairment

## Abstract

As visual impairment (VI) due to adverse drug reactions (ADR) is rare in adults and children, there is an incomplete evidence base to inform guidance for screening and for counseling patients on the potential risks of medications. We report on suspected drugs and the eye conditions found in a national study of incidence of diagnosis of visual impairment due to suspected ADR. Case ascertainment was via the British Ophthalmological Surveillance Unit (BOSU), between March 2010 and February 2012, with follow-up after 6 months. Case definition: any child or adult with bilateral or unilateral visual impairment due to a suspected ADR, using distance acuity worse than Snellen 6/18 (logMAR 0.48) in the better eye (bilateral) or affected eye (unilateral). Anonymized patient information on potential cases was provided by managing ophthalmologists, comprising visual status before and after suspected ADR, ophthalmic condition attributable to the ADR, preexisting eye disease and prescribed medications at the time of the ADR. Permanency and causality of the visual impairment were confirmed by the managing clinician, after 6 months, using the WHO Uppsala Monitoring Committee criteria. Over 2 years, 36 eligible cases were reported of whom 23 had permanent VI. While most cases were due to drugs known to have adverse side-effects, some were unanticipated sporadic cases. Visual impairment due to ADRs is rare. However, with for example, increasing polypharmacy in the elderly, monitoring of ocular ADRs, although challenging, is necessary.

## Introduction

Visual impairment as an adverse side-effect of medication is rare but can lead to considerable individual and societal burden. Acquiring robust data to identify and confirm a relationship between a medication and an uncommon adverse side-effect is challenging.

In the United Kingdom, medications are ‘monitored’ through the Medical and Health product Regulatory Agency (MHRA). All serious *suspected* adverse drug reactions (ADR) and any drug-related side-effect of a new (black triangle) medication (Kelly 2009) are reported using the *voluntary* Yellow Card Scheme (Medical and Health product Regulatory Agency [MHRA); voluntary Yellow Card Scheme [YCS[) to inform an *anonymized* national database. However, as ocular ADRs are classified by eye condition rather than functional impact or vision, estimation of population incidence of visual impairment due to these ADRs is not possible through this source.

We, therefore, carried out a national active surveillance study of incidence of diagnosis of visual loss due to ADRs, through British Ophthalmological Surveillance Unit (BOSU), and have previously published a brief report on the incidence and an evaluation of the national monitoring of ADRs through the MHRA Yellow Card system, based on voluntary reporting of events (Cumberland et al. 2014).

Our study found, as expected, that visual impairment due to ADRs is rare in both adults and children and while the majority of cases were due to drugs known to have adverse side-effects a few were unanticipated sporadic cases. We report here on suspected drugs, eye conditions and clinical detail relating to ADR cases at time of notification and at follow-up, after 6 months. This method of active surveillance has been able to provide otherwise inaccessible information on visual loss due to ADRs, including involvement of some medications previously not known to cause such ADRs.

## Materials and Methods

### Case definition

Any individual (child or adult) with newly diagnosed significant visual loss which is suspected to be due to an ADR to any prescribed medication (topical or systemic) (World Health Organisation[Bibr b18]), to include any of the following: bilateral or unilateral visual impairment due to suspected ADR that is, patient eligible for certification as sight impaired (SI) (partial sight) or severely sight impaired (SSSI) (blind), based on acuity or visual fields, or patient with distance acuity worse than Snellen 6/18 (logMAR 0.48) in the better eye if bilaterally affected or in the affected eye if unilateral, (WHO modified taxonomy).

Patients with new ophthalmic signs and symptoms compatible with an ADR but without significant loss of vision as defined above or patients with raised intra-ocular pressure (IOP) or cataract due to topical or oral steroid treatment (i.e., known and common dose-related side-effect), were ineligible.

### Case ascertainment

Active surveillance was carried out through BOSU over 24 months to February 2012, with 6-month follow-up data collection completed by November 2012. BOSU was established in 1997 and is administered by the Royal College of Ophthalmologists in the UK. The mailing list of 850 Consultant Ophthalmologists and Associate Specialists (hospital-based clinicians) and Senior Lecturers in Ophthalmology (academic clinicians) has been developed and is systematically updated. The aim is to involve every senior doctor who may have clinical responsibility for patients with rare ophthalmological conditions. A monthly BOSU report card, listing all conditions under surveillance, is sent to all those on the mailing list. Return of a card to BOSU, reporting a case, triggers a notification to the study team who send the reporting ophthalmologist a standardized data collection form. There is no direct patient contact.

### Procedures

At notification, information was requested on the patient’s visual status prior and post the suspected ADR, the specific ophthalmic condition attributable to the ADR, preexisting eye disease, all medications being taken at the time of the ADR, and the name of the suspected drug with details of dose, duration. and administration route.

Six months after notification, reporting clinicians were sent a follow-up data collection form. Information on both the permanency of the visual impairment reported and the probability of the causality of the ADR, using the World Health Organisation-Uppsala Monitoring Committee assessment criteria (World Health Organisation 2013) was requested. This allowed sufficient time after notification for completion of diagnostic tests and any potential improvement in vision resulting from dechallenge. Up to two reminders were sent to nonresponding ophthalmologists.

The research ethics committee of the UCL Institute of Child Health and Great Ormond Street Hospital, London, approved the study.

## Results

Of 36 *eligible* cases notified through BOSU between March 2010 and February 2012, 18 were permanent cases (confirmed as permanent visual loss at 6 months) and 13 had temporary visual impairment that is, vision recovered above the eligibility criteria threshold after 6 months. Permanency was not confirmed in 5 cases as 6-month follow-up data were not available. Thirteen of 35 (37%) cases were male (1 case – missing data on sex). There were no children and most cases were over 60 years of age (5 [15%[ between 20 and 40 years, 11 [32%[ aged 41–60 years, and 18 [53%[ at least 61 years). Three subjects prescribed ethambutol (EMB) were of non-White ethnicity (2 Indian and 1 African), and all others were White.

### Ophthalmic conditions resulting from ADR

**Figure 1:**
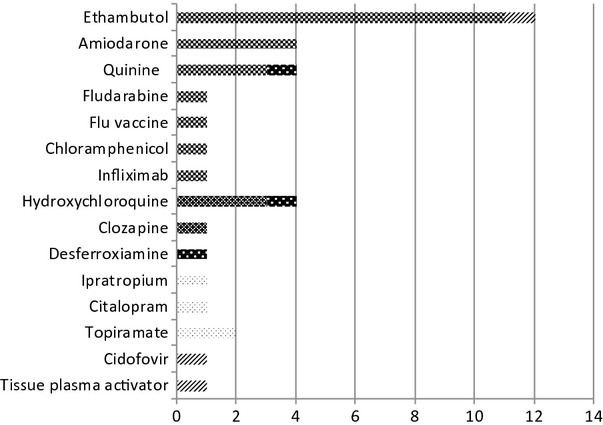
Ophthalmic condition caused by ADR, by suspected drug (*N* = 36). 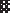, optic neuropathy; 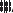, maculopathy; 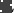, retinopathy; 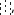, angle-closure glaucoma; 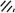, other/missing.

Of the 36 cases, 22 (61%), were reported as having optic nerve disease (optic neuropathy/neuritis/atrophy), 4 had maculopathy, 3 retinopathy, and 4 cases had angle-closure glaucoma. Other conditions included severe anterior uveitis, ocular hypotony, and choroidal body detachments. (Figure1).

### Permanent and temporary cases, by suspected drug

Most reports involved drugs known to have adverse ocular side-effects but there were several other unanticipated medications reported (Panel Table1).

**Table 1:** Clinical detail on notified cases, by suspected drug and permanency status 6 months after notification

Suspected drug	Condition	Prescription reason	Age band	Prior event: VA in better eye	Post event: VA in better eye	Post event: visual field	Post event: color vision	Follow-up: VA in better eye	Follow-up: visual field	Follow-up -color vision	*Abnormal* electro- physiological test results	CSI	LVA	Unilateral or Bilateral	Single/Poly pharmacy	Probability suspected drug cause of ADR
Ethambutol	Optic nerve disease (P)	Atypical Tb	3	N/K	2/60 pinhole	N/T	N/T	3/32	N/T	N/T	PERG PVEP	CSSI	PR	Unilateral Lt A/E	Poly	Possible
Ethambutol	Optic neuropathy (P)	Tuberculosis meningitis	4	6/15	CF	N/T	Abnormal	1/60	N/T	N/T	ERGs – NT PVEP & flVEP	CSSI	CAP	Bilateral	Poly	Possible
Ethambutol	Optic neuropathy (P)	Mediastinal Tb lymph- adenopathy	2	6/9	6/36	<10 deg	Abnormal	6/24	WPD	Abnormal	PERG, FERG & mfERG PVEP & flVEP	CSI	CAP	Bilateral	Poly	Probable/likely
Ethambutol	Optic neuropathy (P)	Not specified	4	CF & Ab VFs	CF	M	Abnormal	6/12	<10 deg	Abnormal	PERG PVEP	CSI	N/A	Bilateral	Poly	Certain
Ethambutol	Optic neuropathy (P)	Tuberculosis meningitis	0	N/K	HM	N/T	N/T	6/24 pinhole	N/T	Abnormal	N/T	CSSI	N/K	Bilateral	Poly	Very probable
Ethambutol	Optic neuropathy (P)	Tuberculosis	2	N/K	CF	WPD	Abnormal	6/18	WPD	Abnormal	PERG PVEP & flVEP	CSSI	N/A	Bilateral	Poly	Probable/likely
Ethambutol	Optic neuropathy (P)	Mycobacterium avium/emphysema	4	N/K	6/9 pinhole	Normal	N/T	6/12 pinhole	WPD	Abnormal	N/T	CD	CAP	Bilateral	Poly	Certain
Ethambutol	Optic neuropathy (T)	Tuberculosis	4	6/6	6/9	<10 deg	Normal	6/9-2	Abnormal	Abnormal	ERGs – NT PVEP & flVEP	N/E	N/A	Bilateral	Single	Probable/likely
Ethambutol	Optic neuropathy (T)	Atypical Tb (following immune-suppression)	2	6/9	<6/18	VF loss	Abnormal	6/6	N/T	Normal	N/T	N/E	N/A	Bilateral	Single	Probable/likely
Ethambutol	Optic neuropathy (T)	Mycobacterium avium	4	Normal	6/18	WPD	Abnormal	6/6	Normal	Abnormal	PERG PVEP & flVEP	N/E	N/A	Bilateral	Poly	Probable/likely
Ethambutol	Optic neuropathy (T)	Pulmonary mycobacterium avium	2	6/5	6/24	Normal	Abnormal	6/5-3	Normal	Abnormal	N/T	N/E	N/A	Bilateral	Poly	Probable/likely
Ethambutol	M (T)		4	M	M	M	M	M	M	M	M	M	M	M	M	M
Hydroxychloroquine	Maculopathy (P)	Systemic lupus erythematosus	1	6/6	6/6-1	M	M	6/6-2	N/T	N/T	Grossly affected PERGs ↓EOGs	N/K	N/K	Bilateral	Single	D/K
Chloroquine Phosphate	Bulls Eye maculopathy (P)	Arthritis	3	logMAR 0.0	logMAR 0.0	N/T	N/T	6/9	<10 deg	Abnormal	PERG PVEP & flVEP	N/E	N/A	Bilateral	Single	Certain
Hydroxychloroquine	Maculopathy (T)	Rheumatoid arthritis	3	6/7.5	6/9	Normal	Abnormal	6/6-2	N/T	Abnormal	PERG & mfERG, EOGs & PVEP	N/K	N/K	Bilateral	Poly	Probable/likely
Hydroxychloroquine	Retinopathy (NC)	Not specified	3	N/K	6/18	<10 deg	M	M	M	M	ERGs & VEP reduced	CSI	CAP	Bilateral	Poly	Probable/likely
Quinine	Quinine toxicity	Muscular cramps	2	N/K	6/12	<10 deg	Normal	6/15	N/T	Abnormal	EDTs show Quinine toxicity	CSSI	CAP	Bilateral	Poly	Certain
Quinine Sulfate	Central retinal artery occlusion resulting in optic atrophy (P)	Muscular cramps	4	N/K	PL	N/T	N/T	6/36	N/T	Abnormal	NT	CSSI	PR	Bilateral	Poly	Certain
Quinine	Optic neuritis (NC)	Intermittent cramps	4	CF; 6/9 pinhole	HM; 6/12 pinhole	M	N/K	M	M	M	Awaiting results	M	M	Bilateral	Single	Possible
Quinine Sulfate	RPE atrophy & retinopathy (NC)	Night cramps	3	6/9	HM	N/K	N/T	M	M	M	FERG PVEP & flVEP	CSSI	CAP	Bilateral	Poly	Possible
Amiodarone	Optic neuropathy (P)	Atrial fibrillation	4	N/K	6/24	N/T	N/T	6/18	<10 deg	N/T	N/K	CSI	CAP	Bilateral	Poly	Certain
Amiodarone	Optic neuropathy (P)	Atrial fibrillation	4	6/9	6/9	N/T	N/T	logMAR 0.18	WPD	Abnormal	N/K	CSI	CAP	Bilateral	Poly	Probable/likely
Amiodarone	optic neuropathy (NC)	Atrial fibrillation	3	N/K	6/5	<10 deg	M	M	M	M	No results reported	CSSI	CAP	Bilateral	Poly	Probable/likely
Amiodarone	Optic neuropathy (NC)	Atrial fibrillation	4	N/K	6/18; 6/9 ph	Abnormal	M	M	M	M	N/K	N/E	N/A	Bilateral	Poly	Probable/likely
Cidofovir	Severe anterior uveitis, ocular hypotony, choroidal ciliary body detachments (P)	Acute myeloid leukemia	2	6/5	CF	N/T	N/T	M	M	M	N/T	CSSI	CAP	Bilateral	Poly	Probable/likely
Clozapine	Bulls Eye maculopathy (P)	Under psychiatric care	0	6/9	6/24	WPD	N/T	6/18	Normal	N/T	N/T	N/E	N/K	Bilateral	Poly	Probable/likely
Fludarabine	Optic nerve and retinal ganglion cell neurotoxicity (P)	Acute myeloid leukemia, MDS trisomy 7	2	N/K	6/36	WPD	Abnormal	6/24	N/T	M	PERG & mfERG PVEP & flVEP	CSSI	CAP	Bilateral	Poly	Probable/likely
Tissue plasminogen activator	Use of tpA in presence of acrylic IOL. Film within IOL (P)	Uveitis	4	6/9	6/9; 6/18	<10 deg	N/T	6/18; HM	<10 deg	N/T	N/K	CSI	N/A	Unilateral	Poly	Possible
Influenza vaccination	Bilateral optic neuritis (P)	Prophylaxis	0	N/K	6/9; CF	<10 deg	Abnormal	6/5-1; 6/24	NAD; Central scotoma	Abnormal; Absent	N/K	N/E	N/A	Bilateral	N/A	Don’t know
Chloramphenicol	Optic neuropathy (T)	Septic Arthroplasty	3	N/K	3/24	WPD	Abnormal	6/9	Normal	Normal	N/K	N/E	N/A	Bilateral	Poly	Certain
Citalopram	Bilateral angle-closure glaucoma (T)	Depression/anxiety	1	N/K	6/5	Normal	N/T	6/6	N/T	N/T	N/T	N/E	N/A	Bilateral	Single	Certain
Desferrioxamine	Retinopathy (T)	Iron overload – transfusions for sickle cell anemia	1	6/6	HM; 6/6	N/T	Normal	6/12; 6/6	N/T	N/T	PERG & FERG VEP – NT	N/E	N/A	Unilateral	Poly	Probable/likely
Infliximab	Lt Optic neuritis (T)	Crohn’s disease	0	N/K	6/5; NPL	N/T	N/T	6/5; 6/5	N/T	Normal	N/K	N/E	N/A	Unilateral	Single	Probable/likely
Ipratropium bromide	Angle-closure glaucoma (T)	Bronchiectasis Pneumo-nectomy	2	N/K	PL	N/T	N/T	6/6	N/T	N/T	N/T	N/E	N/A	Bilateral	Poly	Certain
Topiramate (2 cases)	(T)	M	M	M	M	M	M	M	M	M	M	M	M	M	M	M

Condition: Permanent (P)/Temporary (T)/Not confirmed (NC) i.e., no follow-up information at 6 months. Age band: 0 = 29 up to 40 years, 1 = 41 up to 50 years 2 = 51 up to 60 years, 3 = 61 up to 70 years, 4 = over 70 years. Visual acuity: N/PL, no/perception of light; HM, hand movements only; CF, counting fingers @ 1 m; N/K, not known; M, missing. Visual Field (VF) Test: N/T, not tested; WPD, would preclude driving (but ≥10 degrees central fixation); <10 deg, <10 degrees from central fixation. Color vision: N/T, not tested. *Abnormal* Electro-physiology tests: PERG/fuERG/mfERG, Pattern/Full/Multi electroretinogram ERG; EOG, electro-oculography; PVEP/flVEP, Pattern/Flash Visual Evoked Potential (VEP). CSI (Certification for Sight Impaired): CD, certification deferred; CSI, certified sight impaired; CSSI, certified severely sight impaired; N/E, Not eligible. LVA (Low Vision Aids assessment): CAP, completed – aids provided; PR, planned referral; N/A, not, appropriate; N/K, Not known; M, missing.

### Ethambutol

Overall, 12 patients (median age 68.5, interquartile range [55, 74.5[; 50% males) were reported with EMB-induced optic neuropathy. In all cases, EMB was withdrawn after the ADR event (1 with concurrent withdrawal of Isoniazid). Dosage ranged between 800 mg and 1.2 grammes daily, median 1 gramme (4 cases; dosage not known). For the 7 permanent cases, the median duration of administration was 11 months (range 20 days to 18 months). Using WHO-UMC criteria, 3/12 cases were certain, 6 ‘probable/likely’, and 2 ‘possible’ ADRs due to EMB (missing data for 1 temporary case).

### Hydroxychloroquine and chloroquine phosphate

Two patients, prescribed hydroxychloroquine, for systemic lupus erythematosus and arthritis, had been taking it for 1 and 2 months at 200 mg once and twice a day, respectively. One patient, prescribed a 250 mg daily dose of chloroquine phosphate for several years for arthritis, was reported to have maculopathy with reduced visual fields. One unconfirmed case had retinopathy after taking hydroxychloroquine for more than 8 years concurrently with other medications and it was reported that coexisting renal impairment could have contributed. All cases had severely affected electroretinograms (ERGs) and abnormal (delayed) visual evoked potential (VEPs) indicative of late toxicity.

### Quinine

Two patients prescribed Quinine for night cramps were reported to have had an adverse reaction due to a single large dose, one deliberately self-administered and the other taken in error in combination with alcohol. Although administration errors, these cases are included as they nevertheless caused VI.

One unconfirmed case with coexisting renal impairment had retinopathy and bilateral retinal pigment epithelium atrophy after taking 300 mg quinine sulfate daily, for night cramps, for over 5 years concurrently with other medications. The other patient, with optic neuritis, was continuing to take 300 mg quinine daily as the only effective treatment for night cramps (Mackie et al. 1997).

### Amiodarone

Four patients, prescribed amiodarone for atrial fibrillation were reported to have optic neuropathy. The two confirmed cases (aged 84 and 85 years) were taking several prescribed medications but had stopped taking amiodarone (one after 9 months of a 200 mg daily dose; missing data for second patient). One patient had preexisting cataract in one eye but during the study period required bilateral cataract surgery. All four cases presented with features characteristic of bilateral amiodarone-induced optic neuropathy (Macaluso et al. 1999; Murphy and Murphy 2005) rather than the acute, unilateral visual loss associated with nonarteritic ischemic optic neuropathy.

### Other medications (permanent cases)

A patient requiring tissue plasminogen activator (TPA) for postoperative uveitis following a phacotrabeculectomy and implant of acrylic intraocular lens (IOL), experienced opacification of the artificial lens which the reporting clinician assessed as possibly due to the use of TPA in the presence of an acrylic IOL. After removal of the IOL, visual acuities in both eyes deteriorated leaving the patient with permanent bilateral visual loss, possibly due to maculopathy secondary to inflammation.

A patient with a psychiatric disorder treated with clozapine had developed Bull’s eye maculopathy. Six months after dechallenge, visual acuity had improved to 6/18 in the better eye and visual fields had fully recovered.

A patient with optic neuropathy and probable cortical visual loss had been treated with ten 30 mg/m^2^ doses of fludarabine, in combination with cytarabine, for acute lymphoid leukemia (MDS Trisomy 7). At follow-up, visual acuity had improved to 6/24 in the better eye. At notification the visual fields were reduced and color vision and electrophysiological tests were all abnormal. Interpretation of MRI brain findings indicated fludarabine to be the causative drug.

A bone marrow transplant recipient with severe anterior uveitis, ocular hypotony, and choroidal ciliary body detachments had been given cidofovir for 17 days for a disseminated Herpes Simplex infection. Normal acuities prior to treatment were reduced to finger counting at 1 m at the first assessment and visual fields were restricted. The patient remained visually impaired until their death months later.

A young adult was reported with optic neuritis 3 weeks after influenza vaccination. At follow-up, the visual acuity in the affected eye had improved to 6/24, however, there was a central scotoma and no color vision. At notification the reporting clinician assessed causality by influenza vaccination as possible but after 6 months, uncertain.

### Other medications (temporary cases)

None of these patients had known eye disease or visual loss prior to the ADR and although initially eligible, at follow-up they were ineligible because of improved visual function.

One patient, with unilateral sickle cell retinopathy and abnormal ERGs, had received desferrioxamine (DFO) for 7 days to treat transfusion-related haemochromatosis. The clinician reported reduced acuity, post-treatment, as probable/likely due to DFO.

A patient with Crohn’s disease was reported to have unilateral retrobulbar optic neuritis after a series of infusions of Infliximab. After cessation of Infliximab, and steroid therapy, visual function improved from no perceived light to 6/5 in the affected eye.

A patient with bilateral optic neuropathy had been prescribed 1 g (×4 daily) of Chloramphenicol for 3 months, to treat septic arthroplasty. At initial assessment visual acuities were 3/24 in both eyes, visual fields reduced, and color vision abnormal. Three weeks after dechallenge, visual acuities were 6/9 in both eyes and visual fields and color vision had recovered.

Four patients were reported to have angle-closure glaucoma triggered by drugs known to have a mydriatic effect. One patient had been prescribed a 20 mg dosage of Citalopram for depression and anxiety 2 weeks prior to the ADR. Although visual acuities were normal the clinician was certain an adverse reaction to Citalopram had caused unilateral glaucoma which required laser iridectomy surgery. Another patient, with angle-closure glaucoma, after using an Ipratropium bromide nebulizer, was only able to perceive light in both eyes at first assessment. A phaco/clear lens extraction was carried out so asthma treatment could be restarted and visual acuities recovered. Two patients with acute visual loss due to choroidal effusions and glaucoma, associated with topiramate use for treatment of migraines, recovered visual function on medical treatment.

## Discussion

This was a time-limited national active surveillance study of visual impairment or blindness (unilateral and bilateral cases) due to ADR. As reported elsewhere, these events are rare with an estimated annual incidence of fewer than 4 in 10 million adults and 1 in 100 milion children (Cumberland et al. 2014). Most reported suspect drugs were known to have potential adverse side-effects but this study will increase awareness of some other drugs with potential adverse ocular effects.

Active surveillance through BOSU has been used effectively for numerous studies of rare disorders or events (Shah et al. 2011; Hamblion et al. 2012). Ascertainment of cases via BOSU is high as assessed by indirect methods but cannot be calculated formally in the absence of a second independent source permitting capture/recapture analysis (Stanford 2002). In a clinical setting, both adults and children experiencing visual loss due to a suspected ADR would be expected to present to an ophthalmologist, so we expected to capture a majority of eligible cases. Nevertheless, we recognize that underascertainment or biased ascertainment may have occurred. As reporting by ophthalmologists occurred after visual loss had occurred, details for example, visual acuity before the ADR, are not available for some cases.

Reactions to drugs with known adverse side-effects may have been underreported and/or underreporting may have occurred due to lack of recognition of an event as an ADR either because the specific reaction had not been seen before or because it was unclear the reaction was related to the drug rather than the underlying medical condition. Additionally, 13 of the 17 cases reported with permanent visual loss were taking at least two other prescribed drugs concurrently and identification of the drugs causing the visual impairment was not confirmed. However, most reporting clinicians named a specific suspected drug and graded causality as at least ‘probable/likely’. This level of uncertainty reflects the ‘real-life’ situation of clinical practice.

### Suspected drugs and conditions reported at the time of notification

Most cases (57%) were reported to have optic nerve disease due to antitubercular, antianginal, or antimicrobial agents (Santaella and Fraunfelder 2007), with suspected ADR due to EMB the most common cause of optic neuropathy reported (12 cases in 2 years).

Arguably, more events in those taking prescribed drugs known to induce acute angle-closure glaucoma could have been expected over 2 years as this is not an uncommon reaction with both topical and systemic medications, particularly in elderly patients (Etminan et al. 2012; Lai and Gangwani 2012). However, it is possible that such acute incidents which resolved quickly and/or ADRs to drugs with known side-effects were underreported to this study by clinicians as the study focus was known to be permanent visual impairment.

Hydroxychloroquine and chloroquine have been associated with many toxic effects however, serious toxicity is very rare with recommended low dose levels (Marmor et al. 2002; Tehrani et al. 2008). It is, therefore, notable that the 2 cases, with normal acuities before the ADR event, were reported to have been taking recommended dose levels for only 1 and 2 months. respectively. These cases highlight the critical importance of existing guidelines (Royal College of Ophthalmologists[Bibr b13]), ensuring that the patient is aware of the need to stop treatment and report any change in visual acuity or blurred vision to the prescribing doctor.

Clinicians are regularly faced with requests to screen patients, particularly children, who are on drugs known to cause ADRs. Screening is a burden on patients, their families, and the resources of Ophthalmology departments and is often not appropriate for ADRs where natural history is unknown. The onset of an ADR is not necessarily linear with dosage and/or duration of drug administration or necessarily correlated with reduction in visual function, so screening those on drugs known to have side-effects does not necessarily reduced the incidence or severity of ADRs. Irrespective of whether screening can be done, the key to early recognition of toxicity is informing the patient (or family) and where relevant, the primary care physician, of the potential risks so as to aid early detection and potential withdrawal of the drug to minimize permanent visual loss.

In the UK, there is currently no way to routinely investigate adverse ocular outcomes at a population level to assess the proportion which might be attributable to specific medications. The potential to use primary care databases, such as the Clinical Practice Research Datalink, exists but there are significant challenges to their use in terms of accuracy and completeness of diagnosis of ADR and coding of these events (Ackers et al. 2007). As we have reported elsewhere (Cumberland et al. 2014) the MHRA national system for pharmacovigilance, based on voluntary passive surveillance, did not prove to be as efficient as active surveillance through BOSU. However, the MHRA have recently published new guidance on reporting suspected ADRs in children http://www.mhra.gov.uk/Safetyinformation/DrugSafetyUpdate/CON444290, which specifies that all reactions associated with use of off-label and unlicensed (OLUL) medicines, which are more likely to be implicated in an ADR than authorized medicines (Bellis et al. 2014), should be reported via the Yellow Card Scheme. The MHRA guidance notes also highlight the importance of vigilance in monitoring elderly patients who, for both pharmacokinetic and pharmodynamic reasons, may be more susceptible to developing ADRs. If MHRA guidance is followed and the information collected is fed back to clinical practice, this has the potential to improve routine surveillance in the future.

This study has highlighted the ongoing challenges in monitoring ADRs which underlie the presently incomplete evidence database for recommendations about screening. There were a few ineligible cases, with mild or moderate visual loss, notified to the study which highlights a need to also understand the considerable burden of mild or moderate visual impairment due to ADR, which was not evaluated by this study. Faced with the challenges of voluntary reporting to the national monitoring scheme that we have reported elsewhere, this method of active surveillance has been able to provide otherwise inaccessible information on ADRs and demonstrate clinical scenarios at a level of detail which is informative to practitioners. It was also useful in creating awareness of the previously unknown potential of some medications to cause ADRs.

## Abbreviations

ADR: adverse drug reactions
BL: blind
BOSU: British Ophthalmological Surveillance Unit
DFO: desferrioxamine
EMB: ethambutol
ERG: electroretinogram
IOL: intraocular lens
MHRA: Medicines and Healthcare products Regulatory Agency
OLUL: off-label and unlicensed
SI: sight impaired
SSI: severely sight impaired
SVI: severe visual impairment
TPA: tissue plasminogen activator
VEP: visual evoked potential
VI: visual impairment
WHO: World Health Organisation
YCS: yellow card scheme
